# Supplementary material for: The quality and safety of Rhodiola rosea supplements on the U.S. market: An analysis of biomarkers, heavy metals, and pesticide residues
Source: PLoS One. 2026 Jan 16;21(1):e0341070. doi: 10.1371/journal.pone.0341070 (PMC12810810; doi:10.1371/journal.pone.0341070)
Supplement: Attachment — Submitted filename: 250822 Rhodiola Quality PLOS ONE Response to Reviewers .docx [file pone.0341070.s003.docx]

**Response to Reviewers**

August 22, 2025

Ref: Submission PONE-D-25-18554; The quality of Rhodiola rosea supplements on the U.S. market: An analysis of biomarker molecule, heavy metal, and pesticide contents

Dear Dr. Yaish,

We sincerely thank both reviewers for their careful assessment of our manuscript and the many useful suggestions that they made. Their efforts have improved our manuscript substantially. We have carefully considered all comments and have made appropriate revisions to the manuscript. For each reviewer comment, we provide a detailed response and indicate any corresponding changes made to the manuscript.

As requested, we have modified the manuscript format to adhere to the *PLoS One* guidelines. Both authors agree to full publication of all underlying data related to this manuscript. Regarding our previous use of “data not shown”, we have now prepared a supplementary file that contains the underlying data in support of the relevant statement.

After further review and considering the reviewers’ comments, we have retained anonymity of all products and removed any identifying reference (e.g., specific sources such as Alaskan *Rhodiola rosea*) to keep the focus on the analytical findings.

We have carefully considered all comments and have made appropriate revisions to the manuscript. Please see our point-by-point response below. Reviewers’ comments are copied and listed in **black,** and our responses are listed in **blue**. For each reviewer comment, we provide a detailed response and indicate any corresponding changes made to the manuscript.

We hope these revisions address the reviewers’ comments and demonstrate our commitment to strengthening the scientific contribution of this study.

Sincerely,

Mahtab Jafari, PharmD

Professor of Pharmaceutical Sciences

Director, The UC Irvine Center for Healthspan Sciences

University of California, Irvine - School of Pharmacy and Pharmaceutical Sciences

**Reviewer #1**

Adulteration of botanical ingredients and their products is a big concern for the botanical dietary supplement and related industries. Therefore information about the quality and authenticity of commercial products is important to gather. For this manuscript, the authors have used standard analytical test methods to evaluate the levels of phenolic marker compounds, some heavy metals, and pesticides in 10 commercial samples labeled to contain Rhodiola rosea root/rhizome.

The text is easy to read and follow, and the experimental part is well done. There are a few minor suggested edits, and suggestions for additional publications that may be helpful to include. But overall, the manuscript can be accepted after a few modifications.

REPLY: Thank you so much for this positive feedback.

Line 40: Please add the botanical family

REPLY: The botanical family Crassulaceae was added, as suggested.

Line 45: “is considered an emerging botanical”… I am not familiar with this term. Presumably the authors mean to write that rhodiola is a botanical with growing consumer interest, but this would be an odd way to state this.

REPLY: Thank you for pointing this out. We have removed this phrase.

Line 50: During which time period did the AGR exceed 10%?

REPLY: We have now clarified the CAGR by specifying that *“…in the United States, these supplements have experienced a compound annual growth rate exceeding 10% between 2019 and 2024 [11].”*

Lines 56 and 57: According to one source (Bejar, 2017), “in some geographical areas, the two most frequently used species, R. crenulata and R. rosea, are becoming vulnerable or at-risk (one source uses the terms “threatened” and “critically endangered” when referring to specific areas).” Hence, I don’t think the sentence that R. rosea is not classified as endanegers is accurate. It depends on the location.

REPLY: Thank you so much for this thoughtful comment. We agree with your comment and have addressed this ambiguity by stating that “…*R. rosea* is currently not included in the *International Union for Conservation of Nature*’s *Red List of Threatened Species*.”

Line 67: What do you mean by USP Catalog No: 1602580? Please indicate which USP monograph you refer to.

REPLY: We apologize for this ambiguity. We referred to the US Pharmacopeial Convention’s Certificate for catalog number 1602580, *Rhodiola rosea* root and rhizome dry extract. We have now added the full name to the text.

Line 75: “surveillance of these products, post-market, is sporadic at best,…How do you know that? Please cite some references to support that post-market surveillance is sporadic. I am not aware of such data, but there is a lot of prejudice about what is going on in the industry.

REPLY: Thank you for your question and suggestion. We were indeed imprecise. We have now modified the statement and say that *“…surveillance of these products, post-market, often simply relies on reports of adverse events and subsequent label modifications* *[23]*.” The supporting reference is Li W, Wertheimer A. 2023. Innov Pharm. PMCID: PMC10686678.

Line 89: How did you determine “brand-diversity”. Please specify what criteria you used.

REPLY: We assembled a panel of products, where each product was from a different brand and produced at a different manufacturing site. We now add this information to our selection criteria.

Line 90: Well-regarded distributors…like whom? Please specify which distributors you refer to. And what are your criteria for well-regarded?

REPLY: Thank you for pointing this out. This characterization was not based on published criteria and has therefore been removed.

Line 197: Two additional papers on the frequency of adulteration of botanicals:
- Ichim MC. The DNA-based authentication of commercial herbal products reveals their globally widespread adulteration. Front Pharmacol. 2019;10:1227. 10.3389/fphar.2019.01227
- Orhan N, Gafner S, Blumenthal M. Estimating the extent of adulteration of the popular herbs black cohosh, echinacea, elder berry, ginkgo, and turmeric – its challenges and limitations. Nat Prod Rep. 2024;41(10):1604-1621. [https://doi.org/10.1039/d4np00014e](https://urldefense.com/v3/__https:/doi.org/10.1039/d4np00014e__;!!CzAuKJ42GuquVTTmVmPViYEvSg!OWgisQr61fZZItDRuIRC65n72dxy4ZMbb4HhreB-jzE98QP2Ekm7a_UQuahlnSOZXWcaWNPzFIxjYFk$)

REPLY: Thank you so much, we have now added these references to the manuscript.

Other publications worth considering:
- Bejar E, Upton R, Cardellina II JH. Adulteration of rhodiola (Rhodiola rosea) rhizome and root extract. Botanical Adulterants Prevention Bulletin. Austin TX, ABC-AHP-NCNPR Botanical Adulterants Prevention Program. 2017;1-8. [http://doi.org/10.59520/bapp.bapb/PenB1664](https://urldefense.com/v3/__http:/doi.org/10.59520/bapp.bapb/PenB1664__;!!CzAuKJ42GuquVTTmVmPViYEvSg!OWgisQr61fZZItDRuIRC65n72dxy4ZMbb4HhreB-jzE98QP2Ekm7a_UQuahlnSOZXWcaWNPzBEytHC4$)
- Gemma S, Multari G, Turco L, Gallo FR. A densitometric HPTLC method for the simultaneous quali-quantitative determination of rosavin and salidroside in Rhodiola rosea L. based commercial products: A quick screening by HPTLC-based fingerprint and antioxidant evaluation. J Liq Chromatogr Rel Techn. 2025. 10.1080/10826076.2025.2462263

REPLY: We sincerely thank the reviewer for bringing these references to our attention. We now include the highly relevant Bejar publication in our revised manuscript, as suggested.

Figures 1 and 2 need to be submitted as high-resolution graphs.

REPLY: Thank you for pointing this out. We investigated the resolution of our figures.

The uploaded versions of Figures 1 and 2 have high-resolution. It appears that their resolution changes once embedded in the PDF file. We communicated this issue with the PLOS One editorial staff, and they suggested the following:

*“Since reviewers and the Academic Editor will have access to your File Inventory, they can download the files in their original resolution as necessary while evaluating the manuscript. If the manuscript is accepted, the figures or images in question will be linked in their full resolution. “*

**Reviewer #2**

Introduction:
1. add full botanical name of Rodiola rosea

REPLY: Thank you. We have added the botanical family of *Rhodiola rosea* (Crassulaceae) to the manuscript text.

2. any information on the mechanism of action is missing

REPLY: Thank you for pointing this out. We had refrained from such a statement because the exact mechanism of action of *R. rosea* is still under investigation. Most previous studies centered on possible effects of the biomarker molecules salidroside and rosavins, which are thought to contribute to the adaptogenic effects of the plant. However, not all of *R. rosea*’s biological activities can be attributed to these biomarker molecules, and the benefits are likely imparted by the synergistic contribution of several of the over 140 organic compounds that have been identified in the plant. We very much look forward to future studies characterizing the impact of the various compounds on the plant’s adaptogenic properties. For now, we have added the following text to the manuscript describing the current state of knowledge on the mechanism of action of the plant. *“Some of the adaptogenic effects of the plant, including its antioxidant properties, have been attributed to these biomarker molecules [20, 21], but the beneficial effects cannot be consistently recapitulated with isolated rosavins or salidroside in laboratory settings. Other molecules, such as quercetin-glycosides, have also been identified as contributors [22]. Therefore, the adaptogenic properties are likely caused by the synergistic activity of at least some of the > 100 organic compounds of the plant.*”

3. it will be beneficial to add information about the negative effect of the potential contaminations.

REPLY: Thank you so much for this comment. We have now expressed the dangers of heavy metal and pesticide contamination with the following section: *“Heavy metal exposure can have dramatic detrimental effects on human health, particularly on kidney function [30], while pesticides may cause chronic health conditions, including asthma, cancer, or diabetes [31].”* In addition, we also point out that contamination with other *Rhodiola* species may raise doubts regarding the product’s efficacy.

Materials and methods:
1. It will be beneficial to add if the product sample was a convinience sample or you used any other criteria. When were the supplements purchased?

REPLY: Thank you for pointing out our oversight in characterizing the selection in more detail. All capsules and tinctures were purchased in 2024, and we have now added this information to the text. The samples were bestselling *R. rosea* products on Amazon.com, and included products in different price ranges. We added this description to the text.

2. Why did you choose 7 capsule products and 3 tinctures?

REPLY: The inclusion of tinctures provided better coverage of the different product types on the U.S. market. We selected more capsules than tinctures because more *R. rosea* capsule products are sold on the market, compared to tinctures. We estimated that this small sample of products, which included the current online bestsellers in different price ranges, would provide a good snapshot of the quality of the *R. rosea* products on the U.S. market.

Discussion:
1. it will be very beneficial to add a comment on the biological activity of the supplements based on the different biomarker molecules content.

REPLY: This is a great idea, but it is difficult to provide such an estimate. As outlined above, and mentioned in our discussion, the biological activity of the *Rhodiola rosea* supplements cannot be reliably based on biomarker molecule content alone, and is, instead, a result of complex (and hitherto incompletely characterized) interactions of the many organic compounds of the plant.

Therefore, we largely refrain from speculating on the biomarkers’ biological activity. However, we included in our discussion that “*Our recent lifespan assays in* Drosophila melanogaster *demonstrated comparable life-extending effects between* R. rosea *extracts with lower concentrations of rosavins and an* R. rosea *extract with a higher concentration of rosavins (S1 File). Whether this equivalence extends to other purported benefits of the plant, such as enhanced energy, mental health support, stress reduction, and exercise performance, remains to be determined.”*

*Rhodiola rosea* products are standardized according to their rosavins and salidroside content, which is why we refer to them as biomarker molecules. Our goal was to verify the composition of the products on the market in the U.S., and to estimate their safety by checking for contamination with some of the most important health hazards. A survey of the biological activity of the products or the biomarker molecules was beyond the scope of this study. We wholeheartedly agree that future studies focusing on the biological activity of these products and their biomarkers would be highly informative. These future studies may include assays measuring the biological activities of a standardized amount of each product in cell cultures, as well as physical performance and stress evaluations in laboratory animals.
