# Supplementary material for: The quality and safety of Rhodiola rosea supplements on the U.S. market: An analysis of biomarkers, heavy metals, and pesticide residues
Source: PLoS One. 2026 Jan 16;21(1):e0341070. doi: 10.1371/journal.pone.0341070 (PMC12810810; doi:10.1371/journal.pone.0341070)
Supplement: Attachment — Submitted filename: 251226 PLoS One Rhodiola Response to reviewer.docx [file pone.0341070.s004.docx]

**Reviewer #3**

The manuscript with the title: The quality of Rhodiola rosea supplements on the U.S. market: An analysis of biomarker molecule, heavy metal, and pesticide contents”, the study is valuable, timely, and relevant to public health, pharmacognosy, and dietary supplement quality control. The work is interesting and the work is suitable for the scope of PLOS one. Here are some comments for improving the manuscript.

*Thank you for the kind evaluation of our work!*

1- The title could be changed to Quality and safety of Rhodiola rosea supplements on the U.S. market: variability in biomarkers, heavy metals and pesticide residues

*Thank you for this suggestion. We have modified the title and incorporated most of the requested changes. Since there was no variability in pesticide residues, we changed the title to “The quality and safety of* Rhodiola rosea *supplements on the U.S. market: An analysis of biomarkers, heavy metals, and pesticide residues.”*

2- In the abstract: Add the analytical methods used in the study (e.g., UPLC, ICP-MS, QuEChERS) to improve clarity in the abstract.

*Thank you for this excellent idea. We have now modified the abstract to include these technical details.*

3- in the abstract, the detected concentration range is missing, please adda in the abstract.

*Thank you for this suggestion. We have added the concentration ranges to the abstract, as suggested.*

4- Keywords is missing, authors should add the keywords in the revised version.

*While keywords are not requested or shown in PLoS One’s author guidelines, we now provide five of them.*

5- All abbreviation should be mentioned in the revised manuscript before the introduction section.

*PLoS One’s instructions for authors at* [*https://journals.plos.org/plosone/s/submission-guidelines.com*](https://journals.plos.org/plosone/s/submission-guidelines.com) *recommend to “[d]efine abbreviations upon first appearance in the text.” We have chosen to follow this guideline in our submission. We have also removed some abbreviations that only occurred once in the manuscript.*

6- Line 58, add suitable reference.

*We have expanded the text in the manuscript to clarify the way* Rhodiola rosea *trade regulations were instituted by the* **Convention on International Trade in Endangered Species***, and have added this reference, as suggested by the reviewer.*

7- in materials and methods section: Please specify how many capsules were pooled per product for analysis?

*Thank you for this suggestion. We have added the following sentence to the* **Sample selection and preparation** *section in* **Materials & methods***: “Fill material from 3 – 10 capsules per product was used for the analysis.”*

8- The models of instruments such as ICP-MS, should be mentioned in M& M.

*We have added the instruments used for the ICP-MS (Agilent 7900), the UPLC (Waters Acquity I-class), the GC/MS (Agilent 7880 and 8890), and the LC/MS protocols (Agilent 6400), as requested.*

9- Most of the references of the methods are missing in the materials and methods.

*Thank you for pointing out this omission. We have now included additional specific references from the AOAC compendium used in industrial settings for heavy metal and pesticide detection protocols. Please note that these procedures are routine and standardized industrial protocols used in U.S.-accredited and certified laboratories such as Eurofins.*

10- There is no mentioned about the number of replication in the experiments (duplicate or triplicate).

*Measurements were only performed once, a strategy also applied in similar high-profile publications evaluating biological compounds in dietary products, such as Cohen et al., 2023 (Quantity of melatonin and CBD in melatonin gummies sold in the US. JAMA. 2023;329(16):1401–1402. doi:10.1001/jama.2023.2296) or Crawford et al., 2022 (Analysis of select dietary supplement products marketed to support or boost the immune system. JAMA Netw Open. 2022;5(8):e2226040. doi:10.1001/jamanetworkopen.2022.26040).*

*The analyses reported in our manuscript were performed by Eurofins Scientific. The technical error in all applied methods was precisely controlled by reference standards sourced from qualified vendors, and measurements were automatically repeated when system suitability criteria were not met. Furthermore, all SOPs and associated documentation were tightly followed in each method, minimizing potential measurement errors.*

11- The samples which do not meet the USP minimium limits should be highlighted in the table.

*Thank you for this comment. Please note that the USP minimum limits are set for dried plant roots and rhizomes, as indicated in the Introduction. Those limits do not apply to dietary products containing* Rhodiola rosea*. Therefore, we refrained from implementing the suggested highlights. However, we have now added the advertised biomarker content on the products’ labels (where known) to Table 1, and marked with an asterisk the two capsular products where measured biomarker amounts were significantly lower than advertised.*

12- Figures 1 & 2: If replicates were performed, add error bars.

*As explained in our response to comment #10, replicate analyses were not performed due to the controlled precision of the measurements. Therefore, no error bars are shown.*

13- heavy metals should be in the form heavy metals ions.

*Thank you for this comment. We have rephrased the corresponding section in our manuscript (***Heavy metals and pesticides** *in the* **Results** *chapter) and altered the Y-axis title in* **Figure 2** *to reflect that the ions of heavy metals were detected in our analyses.*

14- Figures need high resolution.

*We agree and hope that our original images fulfill these criteria. We tested our graphs on Newgen Art Analysis, as suggested in PLoS One’s Instructions for Authors, and they passed. We are uncertain why the images appear in such low resolution after upload onto the PLoS One server, and we will work with the editor if this problem persists.*

15- References, some references the journals are abbreviated , other journals in full name, authors should follow the journal instruction.

*Thank you for pointing this out. We have now corrected this oversight.*
